# Application of computed tomography, positron emission tomography-computed tomography, magnetic resonance imaging, endobronchial ultrasound, and mediastinoscopy in the diagnosis of mediastinal lymph node staging of non-small-cell lung cancer: A protocol for a systematic review

**DOI:** 10.1097/MD.0000000000019314

**Published:** 2020-02-28

**Authors:** Longguo Zhang, Fanqi Wu, Rui Zhu, Di Wu, Yao Ding, Zhongmei Zhang, Ya Gao, Yixin Wan

**Affiliations:** a The Second Clinical Medical School of Lanzhou University; b Department of Respiratory, Lanzhou University Second Hospital; c The First Clinical Medical College; d Evidence-Based Medicine Center, School of Basic Medical Sciences, Lanzhou University, Lanzhou, China.

**Keywords:** carcinoma, lymph nodes, magnetic resonance imaging, mediastinoscopy, mediastinum, non-small-cell lung

## Abstract

**Background:**

Ruling out distant metastases, non-small cell lung cancer (NSCLC)treatment depends on the results of mediastinal node staging (N staging). Several diagnostic methods play central roles in mediastinal N staging. This study is intended to evaluate the existing diagnostic methods and report quality, and to search for the best method for staging mediastinal lymph nodes.

**Methods:**

We searched PubMed, Embase, and the Cochrane Library to identify relevant studies, including randomized controlled trials and retrospective studies. These studies report the application of computed tomography, positron emission tomography-computed tomography, magnetic resonance imaging, endobronchial ultrasound, and mediastinoscopy in the diagnosis of mediastinal lymph node staging of NSCLC. The quality of the literature was assessed using the Quality Assessment of Diagnostic Accuracy Study 2. The true positive, false positive, true negative, and false negative of each study was extracted. The corresponding sensitivity, specificity, and other indicators were calculated and the Summary Receiver Operating curve was established. Then, head-to-head and indirect comparison meta-analyses will be conducted.

**Results:**

The results of this study will be published in a peer-reviewed journal.

**Conclusion:**

This study will provide basis for mediastinal lymph node staging of non-small cell lung cancer.

**PROSPERO registration number:**

CRD42019145667

## 1 Introduction

Non-small cell lung cancer (NSCLC), which comprises 85% of all lung cancer cases, is the most commonly diagnosed cancer and the leading cause of cancer death.^[1,2]^ Accurate tumor node metastasis staging in individuals with NSCLC provides adequate information on the local and distant extent of the disease, guides the options for treatment and evaluates malignancy and prognosis. It also avoids futile thoracotomy and surgical intervention, reduces futile treatment and its associated morbidity and cost, thereby improving the quality of life and cost effectiveness.

Ruling out distant metastases, NSCLC treatment depends on the results of mediastinal N staging. In the absence of distant metastases, NSCLC treatment is determined by the results of mediastinal lymph node staging. Methods of mediastinal lymph node N staging include: imaging, such as computed tomography (CT),^[3]^ positron emission tomography (PET) and PET/CT2-deoxy-2-(18F)fluoro-d-glucose positron emission tomography (FDG-PET) or integrated FDG-PET/CT scan^[4]^; needle-based biopsy techniques, such as endobronchial ultrasound transbronchial needle aspiration (EBUS-TBNA)^[5]^ or endoscopic ultrasonography needle aspiration^[6]^ and finally surgical techniques, including mediastinoscopy.^[7]^ These staging methods play an important role in the mediastinal lymph node staging of NSCLC. However, the question of which mediastinal N staging techniques can supply the clinicians with accurate information about the evaluation of malignancy remains to be answered.

As in the mediastinal N staging of lung cancer, the most widely used imaging modality techniques for mediastinal staging are CT and PET-CT, which had poor sensitivity (SEN) and specificity (SPE).^[8–10]^ For patients with potential resectable NSCLC, invasive methods are essential indispensable for mediastinal N staging.^[11]^ Mediastinoscopy has been traditionally considered the gold standard for the mediastinal staging of lung cancer,^[12]^ but it is both invasive and expensive. In recent years, EBUS-TBNA has also been used in lymph node diagnosis of lung cancer because of its advantages of minimally invasive and highly repeatable.^[13]^ Its SEN in staging the mediastinum had been assessed in many studies, with variable results ranging from 75% to 83%.^[14–16]^ Therefore, the diagnostic effect of these techniques may not very ideal. The purpose of this study was to evaluate parameters such as accuracy of CT, PET/CT, MRI, EBUS-TBNA, and mediastinoscopy in the diagnosis of mediastinal lymph node N staging and to discuss its value, safety, and optimal indication.

## 2 Methods

### 2.1 Design and registration

This protocol will be reported according to preferred reporting items for systematic review and meta-analysis protocols.^[17]^ As a part of our project, this review is registered with the International Prospective Register of Systematic Reviews (PROSPERO). The registration number is CRD42019145667.

### 2.2 Search strategy

Computer retrieval databases PubMed, Cochrane Library, Embase. The search time was built until July 29, 2019. The main search terms for these databases are as follows:

(1) “Computed Tomography” OR “CT” OR “PET-CT” OR “SPET-CT” OR “MRI” OR “Magnetic Resonance Imaging” OR “ndobronchial ultrasound” OR “endobronchialultrasonography” OR “EBUS” OR “endobronchial ultrasound-guided”;
(2) “Non-small-cell lung cancer” OR “NSCLC” OR “alveolar carcinoma” OR “pulmonary blastoma” OR “fetal adenocarcinoma” OR “bronchial lung cancer” OR “bronchioloalveolar carcinoma” OR “bronchial alveolar carcinoma”;
(3) “lymph node”;
(4) “sensitivity” OR “specificity”;
(5) (1) AND (2) AND (3) AND (4).

### 2.3 Eligibility criteria

Studies will be included in this overview if meet the following eligibility criteria: Participants: any patient with lung cancer will be included, there are no restrictions on age, race or nationality. Interventions: At least 1 examination method (CT, PET-CT, MRI, EBUS, mediastinoscopy) was used to diagnose mediastinal lymph node staging of non-small-cell lung cancer. Type of studies: systematic reviews including randomized controlled trials and retrospective analysis will be included.

### 2.4 Data extraction

Search the database by subject word, title/abstract. The retrieved literature was imported into Endnote X7, and 2 reviewers screened the literature by title/abstract alone. Then they further screened the preliminary literature according to the full text. Their differences will be resolved by consultation with a third examiner.^[18]^

The other 2 authors will independently use Excel 2013 to establish the “document extraction table” and carry out the extraction of document information. The information includes:

(1) basic characteristics of literature: basic characteristics of literature: research title, author, country, publication date;
(2) study characteristics: number of randomized controlled trials in each group, intervention measures, control measures;
(3) outcome indicators: true positive, false positive, true negative, false negative, and so on.

Any differences between the 2 authors in the extraction of information will be resolved by negotiation between the third author. These 2 authors also need to evaluate the quality of the included literature.

### 2.5 Risk of bias (quality) assessment

The quality of the literature was independently evaluated by 2 reviews. The quality of the literature was evaluated using the Quality Assessment of Diagnostic Accuracy Study 2.^[19]^ The specific evaluation included 4 aspects: patient selection, index test(s), reference standard, flow, and timing. This tool has a total of 14 evaluation points. When the 2 reviews extracted the information of the included documents, they evaluated them according to the scores of “Yes,” “No,” and “Unclear.” The differences between them will be resolved through consultations with the third review.

### 2.6 Strategy for data synthesis

The true positive, false positive, true negative, and false negative of each study were extracted, and the corresponding SEN, SPE, positive likelihood ratio (PLR), negative likelihood ratio (NLR), and diagnostic odds ratio (DOR) were calculated. Statistical analysis was performed using STATA 12.0 and Meta disc (version 1.4). The threshold effect test was performed using Meta disc (version 1.4), and the data were combined when the *P* > .05 was performed by the Spearman rank correlation coefficient. The SPE, PLR, NLR, and DOR were calculated by using the bivariate random mixed effect model. Based on the bivariate model, the SEN and SPE of each study were logit-converted to conform to the normal distribution. The Summary Receiver Operating curve was established from these values and the area under curve and 95% confidence interval were calculated. The Deek funnel plot was used to assess publication bias, and the symmetric funnel plot did not have publication bias.^[20]^ A *P*-value of less than .1 after linear regression analysis indicated potential bias in the study. Analysis of subgroups or subsets: the effect of different lymph node staging on different types of diagnostic SEN.

## 3 Discussion

CT, PET/CT, MRI, EBUS-TBNA, and mediastinoscopy are the primary techniques used for mediastinal N staging in patients with NSCLC. However, these approaches vary in their availability, safety, invasiveness, cost, and reliability. Which one is the optimal manner to evaluate mediastinal lymph nodes metastasis still unknown. Therefore, we will carry out this study by conducting a comprehensive literature search and an indirect comparison between those 5 methods to assess the optimal method. We hope to provide helpful information for clinicians to understand the diagnostic accuracy of the above technologies and to provide the best diagnostic method for mediastinal lymph node staging in patients with NSCLC.

## Author contribution

**Conceptualization:** Fanqi Wu, Longguo Zhang.

**Data curation:** Fanqi Wu, Yao Ding, Zhongmei Zhang, Longguo Zhang, Di Wu.

**Funding acquisition:** Fanqi Wu, Yixin Wan.

**Investigation:** Longguo Zhang, Di Wu, Ya Gao, Zhongmei Zhang.

**Methodology:** Longguo Zhang, Fanqi Wu, Yixin Wan.

**Project administration:** Longguo Zhang, Fanqi Wu, Yixin Wan.

**Resources:** Ya Gao, Yao Ding, Zhongmei Zhang.

**Software:** Ya Gao, Longguo Zhang, Di Wu.

**Supervision:** Fanqi Wu, Yixin Wan.

**Validation:** Fanqi Wu, Yixin Wan.

**Visualization:** Ya Gao.

**Writing – original draft:** Longguo Zhang, Fanqi Wu, Yixin Wan.

**Writing – review and editing:** Longguo Zhang, Fanqi Wu, Yixin Wan.
